# Hospital-Wide Protocol Significantly Improved Appropriate Management of Patients with *Staphylococcus aureus* Bloodstream Infection

**DOI:** 10.3390/antibiotics11060827

**Published:** 2022-06-20

**Authors:** Kawisara Krasaewes, Saowaluck Yasri, Phadungkiat Khamnoi, Romanee Chaiwarith

**Affiliations:** 1 Division of Infectious Diseases and Tropical Medicine, Department of Internal Medicine, Chiang Mai University, Chiang Mai 50200, Thailand; kawisara.k@cmu.ac.th (K.K.); saowaluckyasri@gmail.com (S.Y.); 2 Diagnostic Laboratory, Maharaj Nakorn Chiang Mai Hospital, Faculty of Medicine, Chiang Mai University, Chiang Mai 50200, Thailand; micromedcmu@hotmail.com

**Keywords:** *S. aureus*, bloodstream infection, bacteremia, quasi-experimental study

## Abstract

**Background:***Staphylococcus aureus* bloodstream infection (SA-BSI) causes morbidity and mortality. We established a management protocol for patients with SA-BSI aimed at improving quality of care and patient outcomes. **Methods:** A retrospective pre–post intervention study was conducted at Maharaj Nakorn Chiang Mai Hospital from 1 October 2019 to 30 September 2020 in the pre-intervention period and from 1 November 2020 to 31 October 2021 in the post-intervention period. **Results**: Of the 169 patients enrolled, 88 were in the pre-intervention and 81 were in the post-intervention periods. There were similar demographic characteristics between the two periods. In the post-intervention period, evaluations for metastatic infections were performed more frequently, e.g., echocardiography (70.5% vs. 91.4%, *p* = 0.001). The appropriateness of antibiotic prescription was higher in the post-intervention period (42% vs. 81.5%, *p* < 0.001). The factors associated with the appropriateness of antibiotic prescription were ID consultation (OR 15.5; 95% CI = 5.9–40.8, *p* < 0.001), being in the post-intervention period (OR 9.4; 95% CI: 3.5–25.1, *p* < 0.001), and thorough investigations for metastatic infection foci (OR 7.2; 95% CI 2.1–25.2, *p* = 0.002). However, the 90-day mortality was not different (34.1% and 27.2% in the pre- and post-intervention periods, respectively). The factors associated with mortality from the multivariate analysis were the presence of alteration of consciousness (OR 11.24; 95% CI: 3.96–31.92, *p* < 0.001), having a malignancy (OR 6.64; 95% CI: 1.83–24.00, *p* = 0.004), hypoalbuminemia (OR 5.23; 95% CI: 1.71–16.02, *p* = 0.004), and having a respiratory tract infection (OR 5.07; 95% CI: 1.53–16.84, *p* = 0.008). Source control was the only factor that reduced the risk of death (OR 0.08; 95% CI: 0.01–0.53, *p* = 0.009). **Conclusion:** One-third of patients died. Hospital-wide protocol implementation significantly improved the quality of care. However, the mortality rate did not decrease.

## 1. Introduction

*Staphylococcus aureus* bloodstream infection (SA-BSI) has a mortality rate that ranges from 20 to 40% [1,2]. Poor clinical outcomes, including death, are associated with methicillin-resistant strains, dissemination to multiple organs, the elderly, multiple comorbidities, ICU admission, shock, and inappropriate empiric antibiotic treatment [1,2,3,4,5]. Infectious disease (ID) consultation and adherence to protocols have been shown to improve patient management, e.g., increasing the rate of echocardiography and reducing mortality [6,7,8,9,10].

However, studies have been performed in different hospital settings, and the treatment protocol showed some variations. ID specialists must be involved in the development of treatment protocols. However, ID consultations were not always mandated in the protocols of some studies [6,7,8,9,10]. The value of adherence to treatment protocols, ID consultations, or a combination of both is difficult to determine.

Maharaj Nakorn Chiang Mai Hospital, an affiliated hospital of Chiang Mai University, is a 1400-bed tertiary care referral center in Northern Thailand. There have been 80–90 patients with SA-BSI each year since 2013 (data from the Diagnostic Laboratory, Maharaj Nakorn Chiang Mai Hospital, Chiang Mai, Thailand). The primary care team includes residents and attending physicians who are responsible for patient management and for consulting specialists if indicated. For example, with SA-BSI, the primary care team sometimes manages patients by themselves without ID consultation. To improve patient care, we therefore developed a hospital-wide protocol for the management of patients with SA-BSI.

The primary objective of this study was to determine the 90-day mortality rate of patients who had SA-BSI. The secondary objectives were to determine (1) the recurrent rate of SA-BSI; (2) the rate of appropriate management of SA-BSI, e.g., the rate of investigations for metastatic infections, the rate of follow-up blood culture at 72 h after receiving appropriate antibiotics; (3) the rate of appropriate of antibiotic prescription and factors associated with appropriateness; (4) risk factors for death.

## 2. Results

### 2.1. Demographic Characteristics

**Figure 1:**
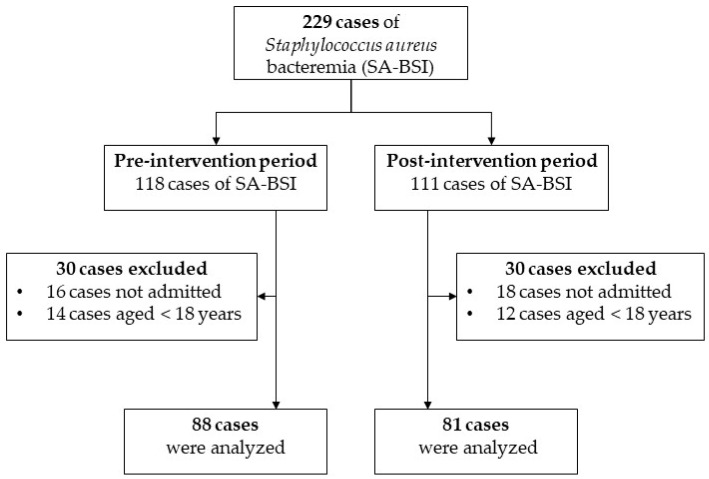
Flow diagram of the cases of *Staphylococcus aureus* bloodstream infection included in the study.

One-hundred and sixty-nine patients who had SA-BSI and met the inclusion criteria were enrolled (Figure 1). The pre-intervention and post-intervention period included 88 and 81 cases, respectively. Patients’ demographic characteristics were generally similar in both periods. In the pre-intervention period, fifty-one patients (58%) were male, and the median age was 64.5 years (IQR 56, 71.5). In the post-intervention period, fifty-one patients (63%) were male, and the median age was 63 years (IQR 50, 72). These characteristics were similar in both periods. Most patients were admitted to the general internal medicine unit. The three most common underlying diseases were hypertension, end-stage renal disease, and diabetes mellitus, respectively (Table 1).

### 2.2. Clinical Characteristics 

Overall, fifty-eight patients (34.3%) had concurrent sites of infection. The common concurrent sites were skin and soft tissue (36 patients, 21.3%), bone and joint (35 patients, 20.7%), and respiratory tract (30 patients, 17.7%) (Table 2). Clinical characteristics were similar in both periods, except that the proportion of patients with shock was higher in the pre-intervention period (36.4% vs. 22.2%, *p* = 0.044). Laboratory data were similar in both periods, except for alanine aminotransferase, which was higher in the pre-intervention period (*p* = 0.023). The proportion of methicillin-susceptible strains was higher in the pre-intervention period (98.9% vs. 91.4%, *p* = 0.029).

### 2.3. Process Measures after Implementation of SA-BSI Treatment Protocol

The appropriateness of the management of patients who had SA-BSI increased significantly in the post-intervention period (Table 3) in terms of both nonpharmacologic and antibiotic management.

#### 2.3.1. Nonpharmacologic Management

First, radiologic imaging was carried out significantly more often in the post-intervention period including echocardiography (70.5% vs. 91.4%, *p* = 0.001) (especially trans-thoracic echocardiography), magnetic resonance imaging (MRI) (10.2% vs. 25.9%, *p* = 0.002), and computerized tomography (CT) scans (5.7% vs. 24.7%, *p* = 0.049). Other details regarding radiologic imaging are shown in Table 3.

Second, follow-up blood culture at 72 h after treatment was performed more often in the post-intervention period (83% vs. 95.1%, *p* = 0.013).

Third, the infectious disease consultation rate was similar in both periods (50% vs. 56.8%, *p* = 0.377). The ID consultation rate for complicated SA-BSI in the post-intervention period was 73.7%; for optional ID consultation for uncomplicated SA-BSI in the post-intervention period, it was 41.9%.

Forth, source control was no different between the pre- and post-intervention periods (34.1 vs. 43.2%).

#### 2.3.2. Pharmacologic Management

##### Antibiotic Prescription

Antibiotics prescribed for the treatment of SA-BSI included cloxacillin, cefazolin, piperacillin/tazobactam, meropenem, imipenem/cilastatin, and vancomycin, as shown in Table 3. Cloxacillin was more likely to be prescribed, ceftriaxone was less likely to be prescribed, and no patients received piperacillin/tazobactam in the post-intervention period. However, rather than looking at specific drugs in detail, we aimed to evaluate the appropriateness of antibiotic prescription, as described in Section “Appropriateness of Antibiotic Prescription”.

##### Appropriateness of Antibiotic Prescription

When looking at each component of appropriateness of antibiotic prescription, we found the following: right drug (72.7% vs. 92.6%, *p* = 0.001), right dose (64.8% vs. 90.1%, *p* < 0.001), right route (85.2% vs. 96.3%, *p* = 0.014), and right duration (52.3% vs. 90.1%, *p* < 0.001) which were higher in the post-intervention period. The appropriateness of antibiotic prescription which included all components was also higher in the post-intervention period (42% vs. 81.5%, *p* < 0.001). The number of patients who received intravenous antibiotics for more than 14 days was significantly higher in the post-intervention period (60.2% vs. 79%, *p* = 0.007).

Factors associated with the overall antibiotic appropriateness from the multivariate analysis were ID consultation (OR 15.5; 95% CI = 5.9–40.8, *p* < 0.001), being in the post-intervention period (OR 9.4; 95% CI: 3.5–25.1, *p* < 0.001), and thorough investigations for metastatic infection foci (OR 7.2; 95% CI 2.1–25.2, *p* = 0.002).

### 2.4. Outcomes Measures after Implementation of SA-BSI Treatment Protocol

Overall, the 90-day mortality was 30% (52 patients). However, mortality attributable to SA-BSI (case-fatality rate) was 20.1% (34 patients). Twenty patients died from other causes: *Acinetobacter baumannii* infections including pneumonia with respiratory failure (four), bloodstream infection (one), and urinary tract infection (one); non-CNS bleeding (four); cardiovascular conditions (two); candidemia (two); lung cancer (one); intracerebral hemorrhage (one); chronic liver disease (one); hospital-acquired pneumonia with respiratory failure from an unidentified pathogen (one).

As the primary objective was to determine overall mortality, we compared overall mortality between the two periods. The mortality rate was 34.1% and 27.2% in the pre- and post-intervention periods, respectively (*p* = 0.329) (Table 3). The patients who died were older, were more likely to be admitted to the medical intensive care unit, and were more likely to have hypertension, dyslipidemia, malignancy, cardiovascular disease, respiratory tract infections, and hypoalbuminemia. On the other hand, patients who survived were more likely to have skin and soft tissue infections and source control (especially drainage), received intravenous antibiotics for at least 14 days, and received appropriate antibiotic prescriptions (Table 4).

The factors associated with mortality from the multivariate analysis were the presence of alteration of consciousness at first presentation (OR 11.24; 95% CI: 3.96–31.92, *p* < 0.001), having a malignancy as an underlying disease (OR 6.64; 95% CI: 1.83–24.00, *p* = 0.004), albumin ≤2.5 mg/dL at first presentation (OR 5.23; 95% CI: 1.71–16.02, *p* = 0.004), and having a respiratory tract infection (OR 5.07; 95% CI: 1.53–16.84, *p* = 0.008). Drainage source of infection was the only factor that reduced risk of death (OR 0.08; 95% CI: 0.01–0.53, *p* = 0.009).

Recurrent SA-BSI was rare (three patients, 1.8%), and thus no further analysis was conducted relating to this outcome.

### 2.5. Post Hoc Analysis

This study was not planned to evaluate the impact of ID consultation in addition to the implementation of a hospital-wide protocol. However, as the rate of ID consultation was not different between the two periods and the overall consultation rate was 53.3%, we further analyzed the impact of ID consultation on the management of patients with SA-BSI. 

From the multivariate analysis, ID consultation was frequently performed in patients who had complicated SA-BSI (OR 6.2; 95% CI: 2.6–14.5, *p* < 0.001). ID consultation frequently led to echocardiography (OR 3.9; 95% CI: 1.2–12.5, *p* = 0.023) and appropriate antibiotic prescription (OR 7.2; 95% CI: 3.1–16.7, *p* < 0.001). 

However, ID specialists were less frequently consulted when a patient was admitted to the intensive care unit (OR 0.3; 95% CI 0.1–0.8, *p* = 0.022).

## 3. Discussion

This study demonstrated that the implementation of a treatment protocol for SA-BSI improved the quality of care. The radiologic imaging rate, follow-up blood culture rate, and appropriateness of antibiotic prescription were significantly increased in the post-intervention period, which is concordant with previous reports [5,7,8,9,11,12,13,14]. This intervention raised awareness among physicians at the hospital of SA-BSI. A clear and concise protocol may guide non-ID physicians to properly manage patients with SA-BSI.

Despite the improvement in the appropriateness of antibiotic prescription in terms of the right drug, right dose, right route, and right duration and thorough investigations to determine whether the patients had metastatic infections, including echocardiography and radiologic imaging (i.e., MRI of the spine and CT scans of the abdomen), we failed to demonstrate a significant reduction in mortality (34% in pre-intervention vs. 27% in post-intervention, *p*-value = 0.329) or recurrent infection. 

Most studies that implemented protocols to treat patients with SA-BSI show improved quality of care and decreased mortality if the protocol was adhered to [5,6,7,9,11,12,13]. The majority of these studies incorporated ID consultation into the protocol. Their results show that ID specialists play an important role and reduce mortality in patients with SA-BSI. However, some studies did not show a significant reduction in mortality rate, even when there was an improvement in antibiotic appropriateness and source control [8,14]. These studies implemented and monitored adherence to the protocol but did not mandate consultations with ID specialists in the post-intervention period, similar to our study. In the authors’ view, along with previous studies [5,7,8,9,11,12,13,14], a hospital-wide protocol that includes ID consultation is recommended.

This study was designed to guide non-ID physicians to manage patients with uncomplicated SA-BSI properly with optional ID consultation. On the other hand, ID consultation is recommended for patients with complicated SA-BSI. However, ID consultation was adhered to in complicated SA-BSI in only 74% of cases in the post-intervention period. The appropriateness of antibiotic prescription was associated with ID consultation, being in the post-intervention period (hospital-wide protocol implementation), and thorough investigation for metastatic infections (with indirectly reflected appropriate treatment duration and source control). Interestingly, ID specialists were less involved in the care of patients that developed SA-BSI in the ICU. The reasons for this need to be further explored.

In this study, the factors associated with mortality were related to host factors and severe infection. These factors included alteration of consciousness at first presentation, having a malignancy as an underlying disease, hypoalbuminemia (albumin ≤2.5 mg/dL) at first presentation, and having a concurrent respiratory infection. The alteration of consciousness may represent an acute brain dysfunction from sepsis [15]. Patients who had a malignancy had a higher risk for infection, subsequent complications, and death [16]. Low albumin may represent a greater severity of illness [17]. Those with a concurrent respiratory tract infection had a high prevalence of septic shock [18]. On the other hand, drainage source of infection can reduce mortality. Patients who underwent source control had a lower mortality rate than patients who did not [19].

In addition, previous studies have reported that factors associated with mortality in SA-BSI consisted of old age [2,3], multiple comorbidities [4], ICU admission [4], previous exposure to antibiotics [4], methicillin-resistant *S. aureus* (MRSA) bloodstream infection [3,4], septic shock [3], liver cirrhosis [3], inappropriate empiric antibiotic treatment, and not receiving an ID consultation [3].

In this study, one-fifth and one-fourth of patients in the pre- and post-intervention periods, respectively, were exposed to antibiotics within 3 months prior to the occurrence of SA-BSI; however, this factor was not associated with treatment outcome. In addition, previous studies have reported that prior exposure to third-generation cephalosporins and fluoroquinolones was associated with MRSA infection [20,21,22,23,24]. We also found a higher proportion of methicillin-resistant strains in patients who had been exposed to antibiotics compared with those who had not (13.2%; 5 of 38 vs. 2.3%; 3 of 131, *p*-value = 0.015). However, nine patients who had been exposed to vancomycin had methicillin-susceptible *S. aureus* (MSSA) BSI. 

The strength of this study lies in the fact that the treatment protocol may be applied to a hospital where infectious disease specialists are not available. Although this study did not demonstrate a death reduction, the patient management improved significantly in terms of the appropriateness of antibiotics, follow-up blood culture, and attempt to discover metastatic infections. 

This study had several limitations. First, the mortality rate appeared to be 34% and 27% in the pre- and post-intervention periods, which were different from the number used for the sample size calculation (40% and 20% for the pre- and post-intervention periods). If this was the case, a larger sample size may be required to detect the difference in the mortality rate. Second, this study was not designed to measure real-time protocol adherence in the post-intervention period, as this was one of the programs for quality improvement and patient safety. However, we performed the process measure including protocol adherence after implementing the protocol as presented in this study. Measuring protocol adherence helped to determine the room for improvement.

One may be concerned about the impact of the COVID-19 pandemic on this study’s results. The first COVID-19 case at the Maharaj Nakorn Chiang Mai Hospital was documented in the third week of January 2020, and the hospital policy then reduced the number of admissions for nonemergency conditions, including nonemergency surgeries and procedures, in March 2020. However, we continued to service patients who required hospitalization. In addition, the number of patients with SA-BSI was similar to the hospital’s annual reports since 2013 (data from the Diagnostic Laboratory, Maharaj Nakorn Chiang Mai Hospital, Chiang Mai, Thailand). Only one patient with COVID-19 in the post-intervention period had SA-BSI, who had an ID consultation and survived. Therefore, if COVID-19 did affect our results, its effects were minimal and similar in both periods.

## 4. Materials and Methods

A retrospective pre–post intervention study was conducted in patients aged ≥18 years old who were admitted to Maharaj Nakorn Chiang Mai Hospital, including the internal medicine, surgery, and orthopedic wards, and had *S. aureus* grow from a blood culture in at least one bottle during the study period. The pre-intervention period was the period one year before implementing the intervention (1 October 2019 to 30 September 2020). The post-intervention period was during the one year after implementing the intervention (1 November 2020 to 31 October 2021). October 2020 was the protocol’s implementation period.

*Implementation of intervention:*

1. ID specialists developed a hospital-wide SA-BSI management protocol. The protocol was adapted from evidence-based data and standard treatment guidelines [20,21,22,23]. The treatment protocol is shown in Supplementary Materials Figure S1;
2. A conference with the primary care team who had a role in the treatment of SA-BSI was held at the Department of Internal Medicine, Surgery, and Orthopedics. The conference described the appropriate management of SA-BSI and explained how to follow the hospital-wide SA-BSI management protocol;
3. The protocol was available at the point of care;
4. We monthly reminded the staff of the treatment protocol in patient care team conferences.

Data collection included demographic and clinical characteristics, treatment outcome, and details of antibiotic prescription.

*Definitions* [24,25]:

1. MSSA is *S. aureus* that is susceptible to methicillin;
2. MRSA is *S. aureus* that is resistant to methicillin.
  *S. aureus* isolates were tested using a disk diffusion method following the Clinical and Laboratory Standards Institute (CLSI) guidelines, M02-A12 (CLSI, 2015), against four antibiotics, namely, erythromycin (15 μg), clindamycin (2 μg), oxacillin (30 μg cefoxitin as a surrogate drug) with a zone diameter ≥22 mm interpreted as methicillin-susceptible (MS) and ≤21 mm as methicillin-resistant (MR), and trimethoprim/sulfamethoxazole (1.25 μg/23.75 μg), (Becton, Dickinson and Company, Franklin Lakes, NJ, USA) [24].
3. Complicated SA-BSI: SA-BSI with one of the following: (1) infective endocarditis; (2) bone and joint infections; (3) metastatic foci of infections, e.g., deep organ abscess; (4) neutropenia; (5) persistent bacteremia after 72 h of appropriate antibiotics; (6) persistent fever after 72 h despite appropriate antibiotic; (7) cardiovascular implantable electronic device (CIED) infection; (8) unable to remove central venous catheter [23].
4. The appropriateness of antibiotic prescription was defined as all of the following: right drug, right dose, right route, and right duration for treatment of SA-BSI accounting for history of drug allergy and intolerance.

*Statistical analysis*

Clinical data are presented as the count (%) or median and interquartile range (IQR) where appropriate. Comparisons of demographic data and clinical characteristics between groups were performed using the Student’s *t*-test, Mann–Whitney U test, chi-square test, or Fisher’s exact test where appropriate. Univariate logistic regression analysis was performed to determine the predictors of fatal outcomes. Variables with a *p*-value < 0.10 in the univariate analysis were then tested in a multivariate logistic regression model using a backward stepwise procedure. A two-sided test with a significance level of *p* < 0.05 was used to determine statistical significance. All statistical analyses were performed using Stata statistical software, version 14 (Stata Statistical Software: Release 14, Stata Corporation, College Station, TX, USA).

*Sample size calculation*

This study was part of a quality improvement and patient safety program. Therefore, we planned to enroll all patients who met the inclusion criteria in both periods. However, to detect the reduction in the mortality rate (the primary outcome) from 40% in the pre-intervention period [1,2] to 20% in the post-intervention period [7], with a power of 80% and a two-sided alpha of 0.05, at least 79 cases per group was required. Therefore, the sample size needed to be 158 cases or more.

## 5. Conclusions

A treatment protocol involving thorough investigations for metastatic infections, follow-up blood culture at 72 h after treatment, and appropriate antibiotic prescription significantly improved the quality of care for patients with SA-BSI. However, the mortality rate did not decrease. A hospital-wide protocol with recommended ID consultation may help to improve patient outcomes.

## Figures and Tables

**Table 1:** Demographic characteristics of patients who had *Staphylococcus aureus* bloodstream infections.

Demographic Characteristics	Pre-Intervention Period (*n* = 88)	Post-Intervention Period (*n* = 81)	*p*-Value
Male	51 (58)	51 (63)	0.506
Age in years (median, IQR)	64.5 (56, 71.5)	63 (50, 72)	0.195
Unit of admission			
▪ General internal medicine	56 (63.6)	49 (60.5)	0.674
▪ Medical intensive care	11 (12.5)	15 (18.5)	0.279
▪ General surgery	11 (12.5)	9 (11.1)	0.780
▪ Surgical intensive care	4 (4.5)	2 (2.5)	0.683
▪ General orthopedic	5 (5.7)	6 (7.4)	0.650
▪ Orthopedics intensive care	1 (1.1)	0 (0)	1.000
Underlying Diseases	79 (89.8)	72 (88.9)	0.852
▪ Hypertension	36 (45.6)	39 (54.2)	0.344
▪ End-stage renal disease	31 (39.2)	35 (48.6)	0.288
▪ Diabetes mellitus	28 (35.4)	29 (40.3)	0.584
▪ Dyslipidemia	17 (21.5)	17 (23.6)	0.787
▪ Malignancy	14 (17.7)	10 (13.9)	0.507
▪ Cardiovascular disease	11 (13.9)	9 (12.5)	0.780
▪ Liver cirrhosis	6 (7.6)	8 (11.1)	0.471
▪ Cerebrovascular disease	4 (5.1)	3 (4.2)	1.000
▪ Chronic lung diseases	2 (2.5)	3 (4.2)	0.671
▪ Intravenous drug use	0 (0)	3 (4.2)	0.108
Prior hospitalization within 90 days	31 (35.2)	32 (39.5)	0.565
Prior antibiotics use within 90 days	19 (21.6)	19 (23.5)	0.772
Beta-lactams			
▪ Oxacillin	1 (1.1)	3 (3.7)	0.284
▪ Amoxicillin or ampicillin	2 (2.2)	2 (2.5)	1.000
▪ Amoxicillin/clavulanic acid	1 (1.1)	0 (0)	1.000
▪ Piperacillin/tazobactam	7 (8.0)	5 (6.2)	0.768
▪ Cefazolin	5 (5.7)	4 (4.9)	1.000
▪ Ceftriaxone	2 (2.3)	6 (7.4)	0.153
▪ Ertapenem	1 (1.1)	1 (1.2)	1.000
▪ Meropenem	6 (6.8)	4 (4.9)	0.749
Other groups of antibiotics			
▪ Vancomycin	6 (6.8)	3 (3.7)	0.499
▪ Fluoroquinolones	3 (3.4)	2 (2.5)	0.717
Medical devices	28 (31.8)	25 (30.9)	1.000
▪ Central venous catheter	17 (19.3)	19 (23.5)	0.512
▪ Urinary catheter	3 (3.4)	3 (3.7)	1.000
▪ Endotracheal tube or tracheostomy tube	5 (5.7)	3 (3.7)	0.459
▪ Intercostal drainage	3 (3.4)	0 (0)	0.247
▪ Automatic implantable cardioverter defibrillator	1 (1.1)	2 (2.5)	0.608
▪ Prosthetic joint	1 (1.1)	1(1.2)	1.000
▪ Permanent double lumen catheter	1 (1.1)	0 (0)	1.000
▪ Tenckhoff catheter	0 (0)	2 (2.5)	0.228
▪ Percutaneous nephrostomy	0 (0)	1 (1.2)	0.479

Data are presented as the count (%), unless otherwise specified.

**Table 2:** Clinical characteristics of patients who had *Staphylococcus aureus* bloodstream infections.

Clinical Characteristics and Laboratory Findings	Pre-Intervention Period (*n* = 88)	Post-Intervention Period (*n* = 81)	*p*-Value
Concurrent site of infection			
▪ Skin and soft tissue	22 (25.0)	14 (17.3)	0.261
▪ Bone and joint	15 (17.1)	20 (24.7)	0.257
▪ Respiratory tract	12 (13.6)	18 (22.2)	0.162
▪ Deep organ abscess	9 (10.2)	15 (18.5)	0.130
▪ Urinary tract infection	13 (14.5)	9 (11.1)	0.503
▪ Infective endocarditis	9 (10.2)	6 (7.4)	0.595
▪ Infectious aortitis	1 (1.1)	2 (2.5)	0.608
▪ Central nervous system	1 (1.1)	3 (3.7)	0.351
▪ Catheter-related bloodstream infection	9 (11.1)	9 (10.2)	1.000
Signs			
▪ Body temperature (°C)	38.4 (37.5, 39)	38.5 (38, 39.2)	0.106
▪ Alteration of consciousness	20 (22.7)	18 (22.2)	1.000
▪ Shock	32 (36.4)	18 (22.2)	0.044
▪ Respiratory rate ≥20/min	63 (71.6)	50 (61.7)	0.174
Laboratory			
▪ Hematocrit (%)	30.3 ± 7.0	30.1 ± 7.4	0.900
▪ Hematocrit ≤30%	48 (54.5)	42 (51.9)	0.726
▪ White blood cell (cell/cu.mm.)	12,575 (7040, 16,435)	12,160 (8550, 16,690)	0.934
▪ Neutrophil (%)	86 (75.2, 91.9)	89 (82.2, 91.5)	0.095
▪ Platelets (/cu.mm.)	178,000 (125,500, 272,500)	190,000 (122,000, 262,000)	0.932
▪ Creatinine (mg/dL)	1.5 (0.8, 4.9)	2.5 (1.0, 6.3)	0.158
▪ Albumin (mg/dL)	3.1 (2.6, 3.6)	3.1 (2.6, 3.7)	0.906
▪ Albumin ≤2.5 mg/dL	13 (14.8)	15 (18.5)	0.609
▪ Alanine aminotransferase (IU/L)	30 (19, 54)	23 (13, 33)	0.023
▪ Total bilirubin (mg/dL)	0.9 (0.4, 1.6)	0.7 (0.4, 1.5)	0.539
Complicated bloodstream infection	30 (34.1)	32 (29.5)	0.466
Blood culture that grew MSSA	87 (98.9)	74 (91.4)	0.029
Vancomycin MIC of MRSA (mg/L)	1 (*n* = 1 patient)	1 (0.5, 1) (*n* = 7 patients)	0.564

Data are presented as count (%), unless otherwise specified. cu.mm., cubic millimeter; mg/dL, milligrams per deciliter; IU/L, international unit per liter; mg/L, milligrams per liter; MIC, minimum inhibitory concentration; MRSA, methicillin-resistant *S. aureus*; MSSA, methicillin-susceptible *S. aureus.*

**Table 3:** Outcomes and management for patients before and after implementation of *Staphylococcus aureus* bloodstream infection treatment protocol.

Outcomes	Pre-Intervention Period Total = 88 Cases	Post-Intervention Period Total = 81 Cases	*p*-Value
90-day mortality	30 (34.1)	22 (27.2)	0.329
Recurrent *S. aureus* bloodstream infection	5 (5.7)	1 (1.2)	0.213
▪ Recurrent within 90 days	0 (0)	0 (0)	NA
▪ Recurrent within 1 year	2 (2.3)	1 (1.2)	1.000
Echocardiography	62 (70.5)	74 (91.4)	0.001
▪ Trans-thoracic echocardiography (TTE)	60 (68.2)	73 (90.1)	0.001
▪ Days of TEE from the first blood culture positivity	5 (3, 8) (*n* = 60 patients)	6 (4, 8) (*n* = 73 patients)	0.209
▪ Trans-esophageal echocardiography (TEE)	6 (6.8)	8 (9.9)	0.471
▪ Days of TEE from the first blood culture positivity	6.5 (6, 8) (*n* = 6 patients)	8 (5, 13.5) (*n* = 8 patients)	0.788
Other radiologic imaging	16 (18.2)	32 (39.5)	0.002
▪ Magnetic resonance imaging	9 (10.2)	21 (25.9)	0.009
▪ Spine	9 (10.2)	20 (24.7)	0.013
▪ Thigh	0 (0)	1 (1.2)	0.479
▪ Computerized scan	5 (5.7)	12 (14.8)	0.049
▪ Abdomen	3 (3.4)	10 (12.3)	0.041
▪ Chest	2 (2.3)	2 (2.5)	1.000
▪ Ultrasonography	6 (6.8)	6 (7.4)	1.000
▪ Arterio-venous fistula	6 (6.8)	3 (3.7)	0.499
▪ Abdomen	1 (1.1)	0 (0)	1.000
▪ Muscle	0 (0)	1 (1.2)	0.479
▪ Joint	0 (0)	1 (1.2)	0.479
Source control	30 (34.1)	35 (43.2)	0.223
▪ Removal of central venous catheter	13 (14.8)	12 (14.8)	0.994
▪ Debridement	11 (12.5)	12 (14.8)	0.661
▪ Drainage	10 (11.4)	12 (14.8)	0.505
▪ Valvular surgery	3 (3.4)	0 (0)	0.247
▪ Removal of automatic implantable cardioverter defibrillator	0 (0)	1 (1.2)	0.479
▪ Removal of percutaneous nephrostomy	0 (0)	1 (1.2)	0.479
Days of intravenous antibiotics, median (IQR)	14 (7, 18)	15 (14, 28)	0.015
▪ Intravenous antibiotics ≥14 days	53 (60.2)	64 (79)	0.007
Afebrile at 72 h after treatment	50 (56.8)	48 (59.3)	0.271
Follow-up blood culture at 72 h after treatment	73 (83)	77 (95.1)	0.013
Intravenous antibiotics for treatment of SA-BSI *			
▪ Cloxacillin	45 (51.1)	57 (70.4)	0.011
▪ Piperacillin/tazobactam	11 (12.5)	0	0.001
▪ Cefazolin	9 (10.2)	4 (4.9)	0.197
▪ Ceftriaxone	10 (11.4)	2 (2.5)	0.025
▪ Meropenem/imipenem	4 (4.5)	4 (4.9)	1.000
▪ Vancomycin	9 (10.2)	14 (17.3)	0.181
Appropriateness of antibiotics use	37 (42.0)	66 (81.5)	<0.001
▪ Right drug	64 (72.7)	75 (92.6)	0.001
▪ Right dose	57 (64.8)	73 (90.1)	<0.001
▪ Right duration	46 (52.3)	73 (90.1)	<0.001
▪ Right route	75 (85.2)	78 (96.3)	0.014
Infectious disease (ID) consultation	44 (50.0)	46 (56.8)	0.377
ID consultation for complicated bloodstream infection	26/33 (78.8)	28/38 (73.7)	0.781
ID consultation for uncomplicated bloodstream infection	18/55 (32.7)	18/43 (41.9)	0.402

Data are presented as count (%), unless otherwise specified. * Two patients and no patients in the pre- and post-intervention periods, respectively, received oral dicloxacillin (*p*-value = 0.498).

**Table 4:** Comparison of the characteristics of patients with *Staphylococcus aureus* bloodstream infections who survived and who died.

Characteristics	Patients Who Survived (*n* = 117)	Patients Who Died (*n* = 52)	*p*-Value
Male	72 (61.5)	30 (57.7)	0.637
Age ≥60 years old	63 (53.8)	40 (76.9)	0.005
Unit of admission			
▪ General internal medicine	78 (66.7)	27 (51.9)	0.068
▪ Medical intensive care	9 (7.7)	17 (32.7)	<0.001
▪ General surgery	17 (14.5)	3 (5.8)	0.104
▪ Surgical intensive care	4 (3.4)	2 (3.8)	1.000
▪ General orthopedic	8 (6.8)	3 (5.8)	0.104
Underlying diseases	102 (87.2)	49 (94.2)	0.279
▪ Hypertension	45 (38.5)	30 (57.7)	0.020
▪ End-stage renal disease	50 (42.7)	16 (30.8)	0.141
▪ Diabetes mellitus	38 (32.5)	19 (36.5)	0.606
▪ Dyslipidemia	17 (14.5)	17 (32.7)	0.007
▪ Malignancy	12 (10.3)	12 (23.1)	0.028
▪ Cardiovascular disease	10 (8.5)	10 (19.2)	0.047
▪ Liver cirrhosis	11 (9.4)	3 (5.8)	0.554
▪ Cerebrovascular disease	4 (3.4)	3 (5.0)	0.677
▪ Chronic lung diseases	2 (1.7)	2 (3.8)	0.588
Prior hospitalization within 90 days	44 (37.6)	19 (36.5)	0.895
Prior antibiotics use within 90 days	27 (23.1)	11 (21.2)	0.782
Medical devices	36 (30.8)	17 (32.7)	0.804
Concurrent site of infection			
▪ Skin and soft tissue	31 (26.5)	5 (9.6)	0.013
▪ Bone and joint	27 (23.1)	8 (15.4)	0.255
▪ Respiratory tract	14 (12.0)	16 (30.8)	0.003
▪ Deep organ abscess	20 (17.1)	4 (7.7)	0.106
▪ Urinary tract infection	13 (11.1)	9 (17.3)	0.269
▪ Infective endocarditis	9 (10.2)	6 (7.4)	0.595
▪ Infectious aortitis	1 (0.9)	2 (3.9)	0.224
▪ Central nervous system	2 (1.7)	2 (3.9)	0.588
▪ Catheter-related bloodstream infection	14 (12.0)	4 (7.7)	0.406
Signs			
▪ Body temperature (°C)	38.5 (38, 39)	38.4 (37.4, 39)	0.102
▪ Alteration of consciousness	13 (11.1)	25 (48.1)	<0.001
▪ Shock	31 (35.2)	19 (23.5)	0.187
▪ Respiratory rate ≥20/min	70 (79.5)	43 (53.1)	0.004
Laboratory			
▪ Hematocrit (%)	31 (7)	28.3 (7.3)	0.023
▪ Hematocrit ≤30%	57 (48.7)	33 (63.5)	0.076
▪ White blood cell (cell/cu.mm.)	12,880(8610, 17,230)	10,690 (6260, 16,420)	0.004
▪ Neutrophil (%)	87 (78.9, 91.5)	89 (78.2, 91.8)	0.967
▪ Platelets (/cu.mm.)	192,000 (130,000, 271,000)	172,500 (87,000, 228,500)	0.094
▪ Creatinine (mg/dL)	1.4 (0.8, 6.6)	1.8 (1.1, 3.3)	0.158
▪ Albumin (mg/dL)	3.3 (2.7, 3.8)	2.8 (2.1, 3.3)	<0.001
▪ Albumin ≤2.5 mg/dL	11 (9.4)	17 (32.7)	0.001
▪ Alanine aminotransferase (IU/L)	24 (15.5, 40.5)	29 (17, 54)	0.394
▪ Total bilirubin (mg/dL)	0.6 (0.4, 1.4)	0.8 (0.5, 2.1)	0.122
Complicated bloodstream infection	42 (35.9)	20 (38.5)	0.750
Source control	53 (45.3)	12 (23.1)	0.006
▪ Removal of central venous catheter	20 (17.1)	5 (9.6)	0.206
▪ Debridement	18 (15.4)	5 (9.6)	0.024
▪ Drainage	20 (17.1)	2 (3.8)	0.024
▪ Valvular surgery	2 (1.7)	1 (1.9)	1.000
Days of intravenous antibiotics, median (IQR)	14.5 (14, 28)	8 (5, 15)	<0.001
▪ Received intravenous antibiotics ≥14 days	97 (82.9)	20 (38.5)	<0.001
Follow-up blood culture at 72 h after treatment	108 (92.3)	42 (80.8)	0.028
Afebrile at 72 h after treatment	76 (65.0)	22 (42.4)	0.126
Appropriateness of antibiotic use	80 (68.4)	23 (44.2)	0.003
▪ Right drug	103 (88)	36 (69.2)	0.003
▪ Right dose	95 (81.2)	35 (67.3)	0.048
▪ Right duration	95 (81.2)	24 (46.2)	<0.001
▪ Right route	104 (88.9)	49 (94.2)	0.396
Infectious disease consultation	66 (56.4)	24 (46.2)	0.217
Being in the post-intervention period	59 (50.4)	22 (42.3)	0.329

Data are presented as the count (%), unless otherwise specified. cu.mm., cubic millimeter; mg/dL, milligrams per deciliter; IU/L, international unit per liter; mg/L, milligrams per liter.

## Data Availability

Some of the data will be shared upon request.

## Supplementary Materials

The following supporting information can be downloaded at: https://www.mdpi.com/article/10.3390/antibiotics11060827/s1, Figure S1: The hospital-wide protocol for *S. aureus* bloodstream infection.
