# Supplementary material for: Hospital-Wide Protocol Significantly Improved Appropriate Management of Patients with Staphylococcus aureus Bloodstream Infection
Source: Antibiotics (Basel). 2022 Jun 20;11(6):827. doi: 10.3390/antibiotics11060827 (PMC9219980; doi:10.3390/antibiotics11060827)
Supplement: Supplementary file 1 [file antibiotics-11-00827-s001.zip › antibiotics-1750640-supplementary.pdf]

Supplementary Materials

Date	Order for one day	Date	Orders for continuation
	❑ CBC<br><br>❑ H/C x II specimens or H/C for CRBSI (if presence of C-line) at **72 hrs.** after start antibiotics (Date: ______________)<br><br>❑ Consult cardio for echocardiogram ALL case at day 5-7th after H/C positive (Date: __________________)<br><br>❑ Remove central venous catheter at Site: _____________<br><br>❑ MRI spine at site___________ IPD urgency if clinical indicated<br><br>❑ CT whole abdomen IPD urgency if clinical indicated<br><br>❑ Consult surgery for drainage if presence of any abscess Site: _______________	**BW** ____<br>**CrCl**____	<u>If pathogen is **MSSA**</u><br>▪ No penicillin allergy →<br>❑ Cloxacillin 2 gm IV q 4 hr.<br><br>▪ Mild penicillin allergy → Cefazolin<br>❑ CrCL ≥ 35 : 2 gm IV q 8 hr.<br>❑ CrCL 11-34 : 1 gm IV q 12 hr.<br>❑ CrCL < 10 : 1 gm IV q 24 hr.<br>❑ HD : 2 gm after HD<br><br>▪ Severe penicillin allergy →<br>❑ Vancomycin 20-25 mg/kg (________ mg) IV loading then ________ mg IV q _____ hr. (Adjust dose based on CrCl and BW)<br><br>❑ Vancomycin trough level before dose 4th (keep trough level 15-20)<br><br><u>If pathogen is **MRSA**</u><br><br>❑ Vancomycin 20-25 mg/kg (________ mg) IV loading then ________ mg IV q _____ hr. (Adjust dose based on CrCl and BW)<br>❑ Vancomycin trough level before dose 4th (keep trough level 15-20)<br><br>QR CODE<br><br>Scan QR code for dose adjustment for vancomycin based on CrCl and BW

Consult ID if patient has any of these conditions

- ❑ Infective endocarditis
- ❑ Cardiovascular implantable electronic devices (CIED) infection
- ❑ Osteomyelitis
- ❑ Septic arthritis
- ❑ Metastatic infection e.g. deep organ abscess
- ❑ Positive H/C at 72 hrs. after proper antibiotics
- ❑ Persistent fever > 72 hrs. after proper antibiotics
- ❑ Neutropenia

↓

If no any of these conditions
Duration of antibiotics IV: 14 days after 1st H/C negative

Figure S1. Hospital-Wide management protocol for patients with *Staphylococcus aureus* bloodstream infections.
